# An educational programme based on salutogenesis theory on childbirth fear and delivery method among nulliparous women: A mixed methods research protocol

**DOI:** 10.1002/nop2.1414

**Published:** 2022-11-06

**Authors:** Safieh Kananikandeh, Farkhondeh Amin Shokravi, Mojgan Mirghafourvand, Shayesteh Jahanfar

**Affiliations:** ^1^ Department of Health Education and Health Promotion, Faculty of Medical Sciences Tarbiat Modares University Tehran Iran; ^2^ Social Determinants of Health Research Center, Faculty of Nursing and Midwifery Tabriz University of Medical Sciences Tabriz Iran; ^3^ MPH Program, Department of Public Health and Community Medicine Tufts University School of Medicine Boston Massachusetts USA

**Keywords:** Educational programme, Fear of childbirth, Salutogenesis, Sense of coherence

## Abstract

**Aim:**

Designing, executing and testing a training intervention based on enhanced concepts of salutogenesis theory for managing fear of childbirth and choice of delivery method among nulliparous women.

**Design:**

A Sequential‐Exploratory Mixed Methods Research.

**Methods:**

In the first phase (qualitative approach), the determinants of childbirth fear among nulliparous women will be explored. In the second phase (systematic review), the factors of childbirth fear among them will be summarized. In the third phase, the content of the educational intervention is developed based on the common factors of childbirth fear obtained from the previous two phases of the study. In the fourth phase (randomized controlled trial), two intervention and the control groups will be compared based on primary and secondary outcomes.

**Discussion:**

Using salutogenesis theory in a few interventional studies on various health areas has produced promising results. Based on the evidence, women had less sense of coherence with a strong childbirth fear. Therefore, developing an effective intervention based on this theory can probably help manage childbirth fear and reduce the costs of any potential consequences.

**Abbreviations**
ST: salutogenesis theory
SOC: sense of coherence
GRRs: generalized resistance resources
FOC: fear of childbirth
W‐DEQ: Wijma Delivery Expectancy/Experience Questionnaire

## 1 INTRODUCTION

Childbirth is a significant occasion in a woman's life (Mehrabadi, Masoudifar, Parvizi, Rakhshani, & Mortazavi, 2020; Onchonga, MoghaddamHosseini, Keraka, & Várnagy, 2020). However, childbirth is not always a pleasant experience for all women, and it may sometimes be a matter of life and death for some (Heidari & Esfandiari Nejad, 2020; Munkhondya, Munkhondya, Chirwa, & Wang, 2020). Women's multidimensional expectations of pregnancy and childbirth can involve a wide range of emotions from joy to fear (Nilsson et al., 2018; Wigert et al., 2020). A common type of fear among women is the fear of childbirth (FOC) (Zarei, Vakilia, & Majidi, 2020).

It has been reported that FOC is associated with the increasing rate of caesarean sections in the United States, Europe, Australia and Iran (Firouzan, Kharaghani, Zenoozian, Moloodi, & Jafari, 2020; Nilsson et al., 2018; Wigert et al., 2020). Although the recommended rate of caesarean section by the World Health Organization is 10%–15%, Iran recorded one of the highest rates of caesarean section in 2017 (50.77%) (Ardakani et al., 2020; Çankaya & Şimşek, 2021; Firoozi, Tara, Ahanchian, & Roudsari, 2020). Hence, this can have short‐term and long‐term consequences for the mother and her child, including uterine infection, fever, bleeding, anaesthesia complications, urinary system damage during surgery, intestinal damage, venous thrombosis, higher expenditure, thromboembolism, postpartum depression, increased need for blood transfusion, placenta prevail, foetal distress syndrome and adverse effects on the infant's immune system (Firoozi et al., 2020; Kananikandeh, 2018; Mortazavi, 2017).

## 2 BACKGROUND

FOC also called tokophobia, or anxiety associated with expected childbirth, is manifested as a phobia, nightmares, physical problems and poor concentration and finally leads to the woman's request for a caesarean section (Andaroon, Kordi, Ghasemi, & Mazlom, 2020; Gönenç, Aker, Güven, & Moraloğlu Tekin, 2020; van Dinter‐Douma, de Vries, Aarts‐Greven, Stramrood, & van Pampus, 2020).

Evidence suggests that one in every five pregnant women has a FOC (Mehrabadi et al., 2020; Mohamamdirizi, Mohamadirizi, & Mohamadirizi, 2018). The prevalence ranges between 5% and 48.9% worldwide (Çıtak Bilgin, Coşkun, Coşkuner Potur, İbar Aydın, & Uca, 2021; Koc, Colak, Colak, Pusuroglu, & Hocaoglu, 2021), between 1.9% and 14% in European countries (Nilsson et al., 2018) and between 5% and 25% in Iran (Andaroon et al., 2020; Yoosefi Lebni et al., 2021). Based on studies, the most common consequences of FOC are infection and bleeding (Zarei et al., 2020), ruptures (Onchonga et al., 2020), eating disorders (Mohamamdirizi et al., 2018), increased risk of dystocia (Çankaya & Şimşek, 2021), increased rate of preterm delivery, avoidance of subsequent pregnancies, maternal and foetal stress, low birth weight and the poor immune system of infants (Hassanzadeh, Abbas‐Alizadeh, Meedya, Mohammad‐Alizadeh‐Charandabi, & Mirghafourvand, 2020).

The possible factors causing FOC are insufficient knowledge, low self‐efficacy, lack of social support, cultural differences, previous history of abortion, the time elapsed from the beginning of pregnancy (Çıtak Bilgin et al., 2021), experiences of previous delivery, delivery method preference, prenatal care and training, educational attainment, age, employment status, economic status, number of pregnancies, primiparity (Serçekuş, Vardar, & Özkan, 2020), place of residence (rural or urban areas) (Badaoui, Abou Kassm, & Naja, 2019) and personality traits of pregnant women (Onchonga et al., 2020). Considering the multidimensional nature of the FOC, a prerequisite to reducing this fear of women is to assess their individual needs (Çıtak Bilgin et al., 2021; Mortazavi, 2017).

Prevention of negative consequences is more emphasize than increasing the welfare of women during the pregnancy period. As evidence suggests, (ST) encourages health professionals to take a health‐focused approach to providing prenatal care for women to help them achieve the ideal health status. By contrast, the theory of pathogenesis focuses on identifying and solving problems (Perez‐Botella, Downe, Meier Magistretti, Lindstrom, & Berg, 2015; Shorey & Ng, 2020). Aaron Antonovsky proposed ST in the 1970s to explain how people cope with stressful situations (Davis, Ferguson, Nissen, Fowler, & Mosslar, 2019; Shorey & Ng, 2020). ST mainly emphasizes the identification of protective factors to maintain health and stability in the face of stressful situations (Hildingsson, 2017). This theory proposes a solution to understanding the health status, from total health to very poor health (Mittelmark et al., 2017; Perez‐Botella et al., 2015). Indicating that one's health status may fluctuate within a continuum throughout their life, ST suggests a shift in the design and delivery of pregnancy care from emphasizing complications and adverse conditions to making efforts to promote the health status of women (Perez‐Botella et al., 2015).

The two main concepts underpinning ST are “Generalized Resistance Resources” (GRRs) and “Sense of Coherence” (SOC). GRRs refer to characteristics of an individual, family, or community that facilitate their ability to deal effectively with stressors. GRRs include materials and goods, physical and biochemical resources, cognitive‐emotional resources, value‐attitudinal resources, interpersonal relationships, and major socio‐cultural aspects (Langeland, Wahl, Kristoffersen, & Hanestad, 2007). The lack or shortage of GRRs generally makes it difficult to deal with stressors (Voogand, Alehagen, & Salomonsson, 2020). SOC also refers to one's ability to use available and potential resources to cope with stressors; it has been stated that one achieves an enduring personality around the age of 30 years (Downe, Agius, Balaam, & Frith, 2020; Hildingsson, 2017). The concept of SOC is measured based on one's understanding of manageability, comprehensibility and meaningfulness (Shorey & Ng, 2020). Comprehensibility refers to one's belief that stressful factors and situations of life are predictable and explainable; manageability indicates that one may feel they take advantage of the necessary resources to respond to the demands of stressors; and meaningfulness refers to the incentives originating from the worth‐investment emotional meanings associated with the challenges of life (Davis et al., 2019; Voogand et al., 2020).

Although previous studies have proven the effects of ST on improving the SOC in participants (Bonmatí‐Tomas et al., 2019; Heggdal, 2015; Heggdal & Lovaas, 2018; Odajima, Kawaharada, & Wada, 2017; Tan, Chan, & Vehviläinen‐Julkunen, 2014; Tan, Chan, Wang, & Vehviläinen‐Julkunen, 2016), this theory has been less employed in developing prenatal care and interventions (Shorey & Ng, 2020). However, Hildingsson et al. and Ferguson et al. showed that SOC is a strong predictor of women's well‐being during the prenatal period, as women with a strong SOC had more positive, calm, and child‐centred attitudes (Ferguson & Davis, 2019; Hildingsson, 2017). Studies have shown that one of the most important pregnancy problems in nulliparous women is FOC (Mohamamdirizi et al., 2018; Munkhondya et al., 2020; Onchonga et al., 2020), which reaches its peak in the third trimester of pregnancy (Koc et al., 2021; Mohamamdirizi et al., 2018). Since ST hypothesizes that there is a wide range of FOC in women, from little fear to severe fear (Yoosefi Lebni et al., 2021), a high SOC level can help women effectively deal with such a stressful situation (Mohamamdirizi et al., 2018). Therefore, this theory can be an alternative to the current pathogenesis model in prenatal care (Mathias, Davis, & Ferguson, 2021).

Studies have shown that strong SOC reduces women's stress during the prenatal period and helps mothers to better manage their conditions and improves their health status and will probably lead to vaginal delivery (Mohamamdirizi et al., 2018; Shorey & Ng, 2020). Nevertheless, some studies indicate that there are some known GRRs during the prenatal period, such as the number of pregnancies, mother's attitude towards delivery, pregnancy planning, delivery method, place of delivery and social support. In contrast, there are also some unidentified GRRs such as religion, culture and beliefs, inner feelings, the meaning of life and preventive health orientation (Ferguson, Browne, Taylor, & Davis, 2016; Hildingsson, 2017; Shorey & Ng, 2020).

Various studies have reported conflicting results about the effects of training interventions on the FOC (Çankaya & Şimşek, 2021; Mehrabadi et al., 2020). According to the national guidelines for holding childbirth preparation classes to encourage women to have a vaginal delivery and reduce caesarean section, a randomized clinical trial was to investigate the effects of childbirth preparation classes programme based on this guideline on FOC in pregnant women in Sabzevar health centers in 2020. The experimental group participated in childbirth preparation classes organized via the Ministry of Health and Medical Education by two project facilitators. The control group received only routine prenatal care from health centre staff. These study results showed that childbirth preparation classes increased the FOC. Therefore, the content of this training should be assessed (Mehrabadi et al., 2020). Despite prospective cohort and clinical trial studies assessed FOC among primiparous women attending healthcare centres and hospitals for prenatal care services. The experimental group attended childbirth preparation class sessions, and the control group received routine care. Results confirmed the importance of childbirth preparation classes in decreasing FOC and increasing the rate of vaginal delivery in primiparous women who attended these classes (Khorsandi et al., 2008; Najafi, Abouzari‐Gazafroodi, Jafarzadeh‐Kenarsari, Rahnama, & Gholami Chaboki, 2016). Moreover, recent findings of a review showed biofeedback, hypnosis, internet‐based cognitive behavioural therapy and antenatal education are reducing levels of FOC (Badaoui et al., 2019).

The high level of FOC among women and, consequently, the high rate of caesarean section in Iran also corroborates the failure of such interventions. Although ample evidence indicates the positive association between SOC and FOC, a few studies about prenatal care have tested ST on healthy individuals (Perez‐Botella et al., 2015; Shorey & Ng, 2020). Therefore, studies on this subject must investigate the importance of this theory and its components in reducing FOC among nulliparous women and encouraging them to undergo vaginal delivery. Considering the relationship between FOC and caesarean section rate and the special attention paid to the potential of ST in reducing FOC and encouraging vaginal delivery in Article 76 of Iranian Sixth Development Plan, this study's goal is to explain nulliparous women's experiences of FOC and review the determinants of FOC among nulliparous women to identify the main factors causing FOC and describe the concepts of ST in this regard. This study aims to design, execute and test a training intervention based on enhanced concepts of ST for managing FOC and choice of delivery method among nulliparous women.

## 3 METHODS

### 3.1 Design

**FIGURE 1:**
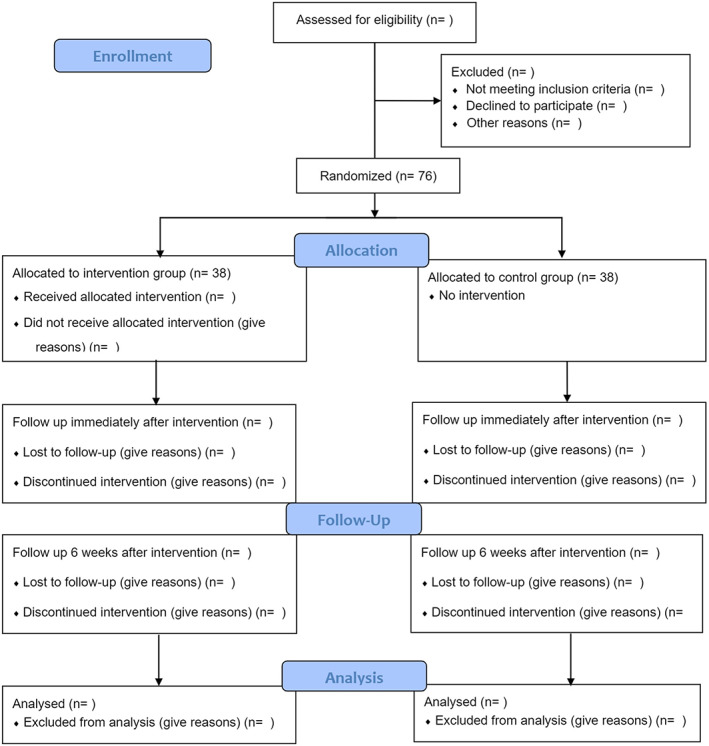
CONSORT flow diagram of the study randomized controlled trial protocol

This mixed‐methods research based on a sequential‐exploratory design will be conducted in the following three phases:

1. The first phase is dedicated to a qualitative study.
2. The second phase is a review study.
3. In the third phase, the content of the training intervention will be developed based on the common factors of FOC obtained from the first and second phases of the study.
4. The fourth phase is a randomized controlled trial (RCT) that aims to investigate the effects of the training intervention on nulliparous women (Fig. 1 Diagram of the study).

It is worth noting that mixed‐methods research, focusing on the meaningful integration of quantitative and qualitative data, can provide a deep understanding that a quantitative or qualitative approach alone may lack. A sequential‐exploratory design is appropriate when the subject is less studied, and there is little information about the relevant components and how to measure its essential variables. In the sequential‐exploratory study, qualitative and quantitative data collection and analysis are in the first and second phases, respectively. Finally, both qualitative and quantitative analyses are interpreted together (Heigham & Croker, 2009; Mohammadpour, Sadeghi, & Rezaei, 2011).

In this study, the content of the training intervention will be developed based on the common factors of FOC obtained from the qualitative and review phases of this study. In the fourth phase, educational intervention is implemented. Finally, qualitative and quantitative analyses interpret together.

### 3.2 The goals of the research three phases are as follows

#### 3.2.1 Qualitative study

Explaining nulliparous women's experiences of determinants of fear of vaginal delivery.

#### 3.2.2 Review study

Identifying the determinants of FOC among Iranian nulliparous women.

#### 3.2.3 Quantitative study

Determining the effect of the training intervention on FOC and delivery method.

### 3.3 Objectives

- Comparing the measures of central tendency and dispersion of FOC among nulliparous pregnant women in control and intervention groups.
- Comparing the rates of choosing vaginal delivery among nulliparous pregnant women in control and intervention groups.
- Comparing the post‐delivery rate of choosing vaginal delivery among nulliparous pregnant women in control and intervention groups.
- Comparing the measures of central tendency and dispersion of SOC among nulliparous pregnant women in control and intervention groups.

### 3.4 Research questions

#### 3.4.1 Qualitative study

Is it possible to reduce the FOC in nulliparous pregnant women by explaining the determinants of FOC and managing them using ST?

#### 3.4.2 Review study

What are the determinants of FOC among nulliparous women?

#### 3.4.3 Quantitative study

- Is training intervention effective in reducing FOC among Iranian nulliparous women?
- Is the training intervention effective in increasing the choice of vaginal delivery among nulliparous women?
- Is the training intervention effective in increasing the SOC mean score among nulliparous women?

### 3.5 First phase: a qualitative exploration

#### 3.5.1 Approach to interviewing and Inclusion criteria

The qualitative data in this phase will be collected using in‐depth semi‐structured interviews with open‐ended questions. The participants will select from nulliparous women in the third trimester of pregnancy referring to the health centres of Bostanabad city, East Azarbaijan province, Iran based on household records. Because, at this time, they are worried about their delivery method, and FOC reaches its peak (Koc et al., 2021; Mohamamdirizi et al., 2018). In addition, they will be selected from different age groups, education attainments, occupational status and socioeconomic status in order to obtain broader codes. Also, participants include doctors, midwives and those involved in making decisions about the delivery method. The participants will be selected using the purposeful sampling method. Recruitment for this study will be via a telephone call. Determining the sample size will depend on the data saturation. Sampling will continue until the data is saturated. Finally, several other interviews will do for accuracy.

#### 3.5.2 Data collection

Face‐to‐face individual interviews will use to collect data. The interviews will be conducted, if possible, from October 2021 to July 2022 in an accurate, quiet, convenient and comfortable environment. Participants will determine the time and place of the interview. The interviews will do by the first author, a Ph.D. candidate in health education and health promotion. She had passed women's health and qualitative research methodology courses before this study. Only the interviewer and participant will be present at the time of the interviews.

The interview questions are developed based on the framework and concepts of ST before performing the qualitative phase in a single session with the researchers. After drafting the questions, a few pilot interviews will conduct to check whether the goal of the qualitative phase can be met with these questions or not. If necessary, the researchers will review and rewrite the interview questions in another session. Eventually, an interview guide will develop for this study.

The interview will begin with the warm‐up question. The first question is: “You know there are different delivery methods; which method did you choose?”. Subsequently, the other questions of the interviews will ask. For example, “Why are you afraid of vaginal delivery?” (comprehensibility), “Can you tell me about how you feel from the moment of vaginal delivery?”(meaningfulness), “How do you react when you are afraid of vaginal delivery?” (manageability), and “How do you get information related to the delivery method” (GRRs). It is worth noting that the sequence of research questions will be different according to the answers provided by the participants to the previous question. Finally, while thanking the study participant, we remind the participant to add specific content if desired and will be asked permission for another interview if needed.

Before beginning the interview, consent will obtain from all participants. They will be assured about voluntary participation in the study, to leave the study at any time if they wish and to keep their personal information confidential in the publication of the results. At first, the researcher introduces herself and her resume. Then the aim, necessity of the study, and the interview process will be stated for participants. All interviews will record using a voice recorder with the participants' agreement. Moreover, notes will be taken during the interview whenever necessary through observation. Then they will be transcribed in Farsi and translated into English for qualitative analysis. Any identifiable personal information of the participants in the transcripts will delete before data encoding.

#### 3.5.3 Credibility and trustworthiness

The accuracy of qualitative findings will be investigated using four criteria of credibility, dependability, transferability and confirmability (Iman & Noushadi, 2011). To increase the credibility of findings, which refers to the confidence that can be placed in the truth of the research findings, comments made by participants, confirmation of research findings by them and data interpretation by the researcher, the methods such as continuous interaction with participants, random sampling, the establishment of intimate and trust‐based relationships with participants to encourage them to answer the questions more honestly, frequent meetings between the researcher and the project manager, reflective interpretation and member check will be used. To ensure the dependability of findings, which represents the stability of findings over time and under similar conditions, the audit trail method (providing a complete set of notes on decisions made during the research process, research team meetings, reflective thoughts, sampling, research materials adopted, the emergence of the findings and information about the data management) will be employed. To increase the transferability of findings, which shows the degree to which the results of qualitative research can be transferred to other contexts or settings with other respondents, an extensive description of the participants and the research process will be provided. Finally, to increase the confirmability of findings, which refers to the degree the research findings reflect the participants' responses, experiences and ideas rather than the researcher's characteristics, preferences, biases or views, the audit trail method (providing a complete set of notes on decisions made during the research process, research team meetings, reflective thoughts, sampling, research materials adopted, the emergence of the findings and information about the data management) will be used (Connelly, 2016; Cope, 2014; Shenton, 2004).

#### 3.5.4 Data analysis

To analyse the data, the qualitative content analysis approach using Kyngäs and Elo methods will be used. This method suggests three phases of preparation (open coding), organization (class creation) and reporting (abstraction) (Elo & Kyngäs, 2008). Written interviews are read frequently before open coding to gain a general understanding of the interviews. In the open coding phase, code is assigned to the semantic units of the code while reading the text using MAXQDA software. In the class creation phase, the codes are compared and classified according to their differences and relationships. In the abstraction phase, in addition to the classes related to the components of ST, new classes will be named based on the codes and content of the subclasses.

### 3.6 Second phase: Systematic review

#### 3.6.1 Search strategy and inclusion criteria

The review study of this research will be performed based on the 2020 PRISMA checklist (Preferred Reporting Items for Systematic Reviews and Meta‐Analyses) (Moher et al., 2009). Accordingly, all Persian and English papers related to published cross‐sectional studies about FOC in nulliparous women will be reviewed from 2000 to 2020 on WOS (Web of Sciences), ScienceDirect, Scopus, PubMed, Cochrane Library, ProQuest, and Persian databases, including Scientific Information Database (SID), Irandoc, and Magiran. The keywords “FOC,” “factors causing FOC,” “nulliparous women,” “delivery method,” “educational programme” and “salutogenesis theory” will be searched to find the relevant papers.

#### 3.6.2 Selection of studies

The information of all papers will be inserted into Mendeley and two independent peer‐reviewers who review the title and abstract of papers to check their relevance to the study subject. The full paper of the selected studies will be qualitatively reviewed to select the best studies that suit the inclusion criterion and remove the inappropriate and irrelevant papers.

#### 3.6.3 Data extraction and quality appraisal

The quality of finalized cross‐sectional studies will be evaluated using the STROBE (Strengthening the Reporting of Observational Studies in Epidemiology) Checklist (Von Elm et al., 2015). This 22‐part checklist evaluates different sections of a paper and gives them a score ranging from 0 to 44; the articles that gain a score lower than 15 will be excluded from the study. Finally, the results of reviewing the selected papers will be presented in a descriptive table that contains title, authors, year of publication, type of study, objective of study, sample size, data collection tools, research setting, findings related to the factors causing FOC and the STROBE Checklist score.

### 3.7 Third phase: Training content design

The content of the training intervention will be developed based on the common factors of FOC among Iranian nulliparous women from the first and second phases of the study.

### 3.8 Fourth phase: Randomized controlled trial

The fourth phase of this study will be performed, and its results will be reported based on the CONSORT (Consolidated Standards of Reporting Trials) 2010 checklist (Schulz et al., 2011).

#### 3.8.1 Study design, participants and setting

In this educational trail RCT, the statistical population consists of all nulliparous women visiting comprehensive health service centres of Bostanabad.

The inclusion criteria are being nulliparous, being at gestational age 6 to 12 weeks (The first trimester of pregnancy is selected to better manage the FOC from the beginning of pregnancy and follow‐up to maintain the intervention for 6 months during pregnancy), being 18–35 years old, having a high school diploma or higher educational degree, no history of depression or long‐term medical illness in the past, singleton pregnancy, ultrasound confirmation of foetal health, no history of miscarriage or stillbirth, no contraindications for vaginal delivery during the study until the end of pregnancy, not being graduated in midwifery or relevant majors and willingness to participate in the study. The exclusion criteria are also emergencies requiring preterm delivery, indications for caesarean section, a history of caesarean section, a history of infertility, irregular attendance in training sessions, refusal to complete the questionnaire and no access to the participant for any unpredictable reason.

#### 3.8.2 Sampling method

First of all, a list of nulliparous women who met the inclusion criteria will be extracted from all health centres of Bostanabad. The sample size for each centre will be determined, and then some women will be randomly selected from each centre. The selected women will be contacted through their contact information to be briefed on the research objective and procedure and then be invited to participate in the study. Sampling will continue until the predetermined number of participants enters the study.

#### 3.8.3 Randomization

The participants will be assigned to two parallel groups of control and intervention using blocked randomization with blocks of 4 and 6. For allocation concealment, the names “Control” and “Intervention” will be written on two separate pieces of paper by a person who will have no role in sampling and data analysis. The pieces of paper will be put inside numbered identical opaque envelopes. Each participant will be given one of the envelopes by the health staff in each centre to be assigned to one of the groups. All participants who will take the measurements will be blinded to the assigned study groups. In the final phase of the study, the intervention will be performed by the researcher.

#### 3.8.4 Intervention

Based on successful evidence in this area (Bonmatí‐Tomas et al., 2019; Heggdal, 2015; Heggdal & Lovaas, 2018; Odajima et al., 2017; Tan et al., 2014, 2016), the training intervention will be performed in four sessions of at least 30 min by using appropriate audio‐visual training methods and tools to investigate each of the common factors of FOC obtained from the qualitative and review phases of this study. It is noteworthy that there would be changes in the number and duration of sessions based on the content obtained from the first and second phases of the study. Training sessions will be held in person probably in four groups of six and two groups of seven. However, the size of the groups may be determined depending on the conditions of the study group, the location of sessions and the number of samples selected from each centre. If the COVID‐19 pandemic reaches a stable state, the training sessions will be held under appropriate physical distancing and health protocols.

For the ease of group management and convenience of participants, training sessions will be held once a week for each group in the relevant, comprehensive health centre. After obtaining written consent from the participants to participate in the study, they will complete the questionnaires in the pre‐test phase. The person who will lead the sessions is a Ph.D. student in health education and health promotion.

Training sessions are taking place based on the priority of the educational domain (cognitive, emotional and behavioural). The first training sessions are based on the cognitive domain includes increasing comprehensibility and identifying GRASs, the next session based on the emotional domain includes increasing meaningfulness, and the last session based on the behavioural domain includes increasing manageability, respectively.

Since the qualitative phase of the study is conducted with a directed content analysis approach, the codes obtained from this phase are categorized as themes based on the components of ST. If these codes are common with the factors of the review phase of the study, the training content will be designed for this theme.

For other codes that are not included in these components of ST and form a new theme, if they are common with the factors obtained from the review phase of the study, the training content will be designed for the new theme.

Hence, the training content will be developed based on the common factors of FOC obtained from the qualitative and review phases of this study and it is provided through various training methods and tools according to the relevant training domain. Details of the training intervention are in Table 1.

**TABLE 1:** Training intervention

Training purpose		Training domain	Training method	Training tools
SOC	Increasing comprehensibility	Cognitive	Lecture, Group discussion, Question‐and‐answer sessions and group learning	Book, Poster, Brochure, Photographs, Educational videos and slides
	Increasing manageability	Behavioural	Problem‐solving, Conversations about positive experiences and group discussions	Memory retelling, Problem‐solving workshop, Group discussions on social media platforms, Using pictures and videos using the effective role of the foetus with motivating messages
	Increasing meaningfulness	Emotional	Brainstorming, Share feelings and ideas	Written and oral presentation of participants' values, motivations, needs and desires concerning pregnancy and childbirth, on a piece of paper and discussion
Identifying GRASs		Cognitive	Group discussion, Question‐and‐answer sessions and group learning	Description and sharing of personal skills of participants with others, Group discussions on social media platforms, Drawing the pictures of family members (for recognizing participants' roles and support levels in the family).
New component		?	?	?

Participants in the control group will receive the routine training provided by the staff of comprehensive health service centres. In addition to the routine training, those in the intervention group will also participate in the training sessions. A text message will be sent to participants before each session to remind them about the session's time and place. Participants will also be given a contact number for asking their possible questions.

The research team will try to finish all the training sessions before the 16th week of pregnancy and conduct the posttest in two phases (the first one immediately after the intervention and the second one during the third trimester of pregnancy [6 months after the intervention]). The participants will be asked to complete the questionnaires once again in the posttest and explain the delivery method and the reason for choosing it after childbirth. Until the end of the study, we will be in touch with the participants to the retention and follow‐up completely in the WhatsApp group.

#### 3.8.5 Data collection

The measurement tools of this study are a demographics questionnaire, the Wijma Delivery Expectancy/Experience Questionnaire (W‐DEQ), Antonovsky's sense of coherence scale (SOC), and a choice of delivery method checklist. All these tools will be completed by participants themselves (self‐reporting). It is noteworthy that there would be changes in the measurement tools depending on the component obtained from the first and second phases of the study.

The W‐DEQ was developed by the Sweden Klaas Wijma in 1998 (Wijma, Wijma, & Zar, 1998). The W‐DEQ is a short, practical and common tool for evaluating FOC (Badaoui et al., 2019). This tool consists of 33 items that are scored based on a 6‐point Likert scale (from 0: very much to 5: not at all). The total score on this scale ranges between 0 and 165, and higher scores indicate higher levels of FOC. As the cut‐off point of this scale is 85, scores over 85, under 38, between 38 and 65 and between 66 and 84 represent clinical fear, mild fear, moderate fear and severe fear, respectively.

The 13‐item Antonovsky's sense of coherence scale (SOC‐13) measures comprehensibility (5 items), manageability (4 items) and meaningfulness (4 items) (Antonovsky, 1993). The items are scored based on a 7‐point Likert scale and the total score on this scale ranges between 13 and 91. A score within the ranges 13–60, 61–75 and 76–91 indicates low, moderate and high levels of SOC, respectively (Voogand et al., 2020).

#### 3.8.6 Validity and reliability

Andaroon et al. (2020) and Mortazavi (2017) have localized and confirmed the validity and reliability of W‐DEQ in Iran (Mohamamdirizi et al., 2018; Mortazavi, 2017). The validity and reliability of Antonovsky's SOC‐13 have also been confirmed by Mohammadzadeh et al. (Mahammadzadeh, Poursharifi, & Alipour, 2010).

Considering the cultural features of the study area, the reliability of both tools will be assessed by the measurement of internal consistency using Cronbach's alpha and temporal stability measurement using the intraclass correlation coefficient (ICC).

#### 3.8.7 Outcome variables

Reduced FOC and the selection of vaginal delivery are the primary outcomes and SOC is considered the secondary outcome of the training intervention in this study.

#### 3.8.8 Sample size

**FIGURE 2:**
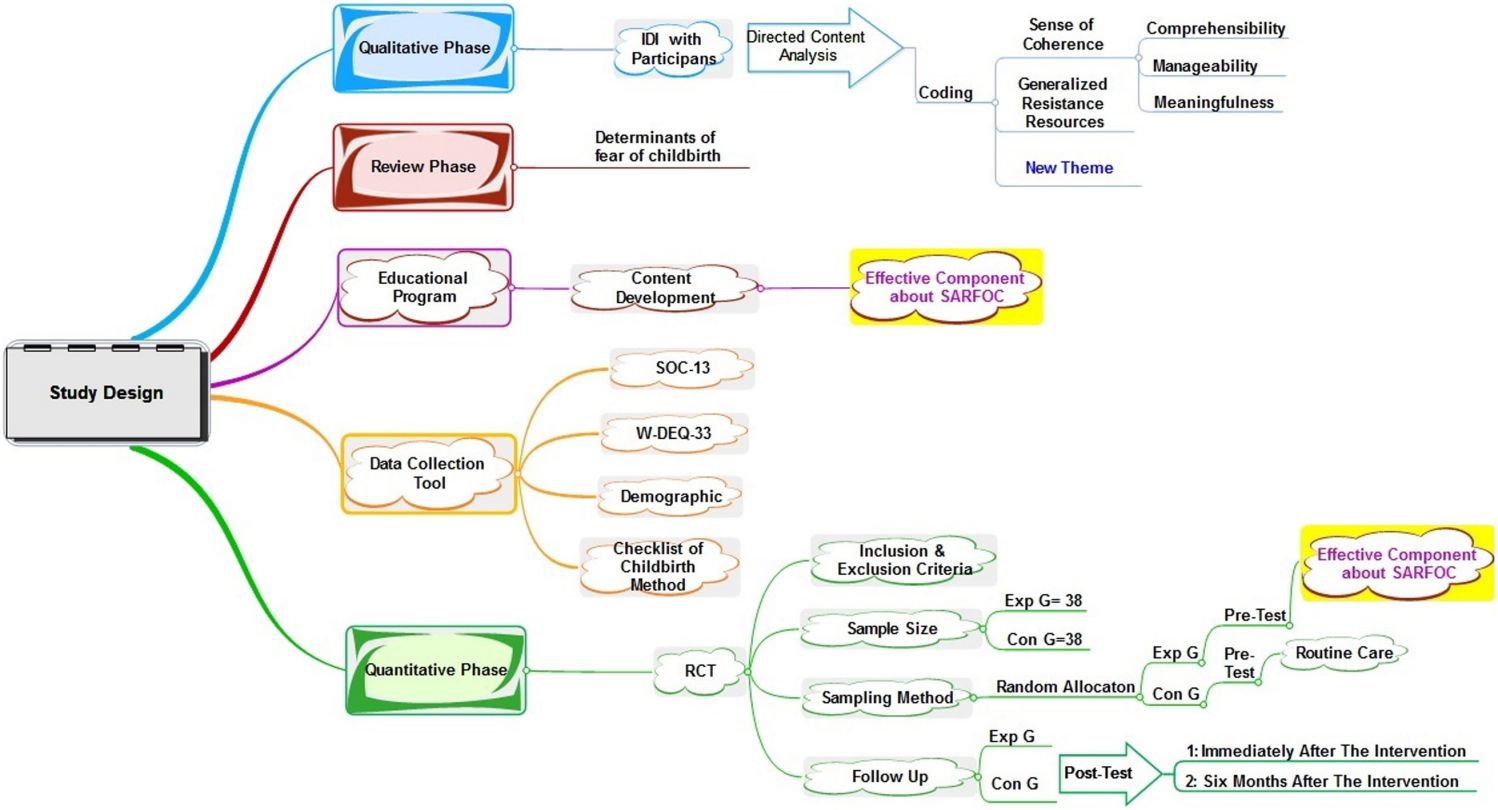
Diagram of the study

Based on the Pukak formula and assuming a confidence coefficient of 95%, a test power of 80%, mean values (±*SD*) of 23 (±4.8) and 20 (±3.7) (related to “meaningfulness” in a similar study conducted based on ST [Tan et al., 2016]), and an attrition rate of 20%, the sample size for each group will be determined to be 38. A flow diagram of the randomized controlled protocol is shown in Fig. 2

$$n=\frac{{\left(Z_{1-\frac{\alpha }{2}}+Z_{1-\beta }\right)}^{2}\left(S_{1}^{2}+S_{2}^{2}\right)}{{\left({\bar{X}}_{1}-{\bar{X}}_{2}\right)}^{2}}$$

#### 3.8.9 Data analysis

After checking the normal distribution of data by the Kolmogorov–Smirnov test, the data will be statistically analysed in SPSS.

The participants' demographics and clinical information will be described by frequency distribution and percentage for qualitative data and mean and standard deviation for qualitative data.

The groups' pre‐ and post‐intervention mean scores of SOC and FOC will be compared by the independent sample *t* test and ANCOVA, respectively. In addition, analysis of variance with repeated measures based on the Greenhouse–Geisser test will be employed to compare the pre‐ and post‐intervention mean scores of SOC and FOC in each of the two groups.

The two groups will also be compared before and after the intervention using a chi‐square test to select the delivery method.

It is noteworthy that nonparametric tests will be used if the data are not normally distributed. The significance level in all tests will be 0.05. If there is missing data, the missing data will be handled using statistical methods.

#### 3.8.10 Ethical considerations

The Medical Research Ethics Committee has approved the research protocol. Before the study begins, a consent form will be obtained from all participants by the researcher, and they will be assured that they will be free to leave the study at any phase they desire. They will also be assured that their personal information will be kept confidential, and the data will be analysed anonymously. The study authors adhere to the Standard Protocol Items: Recommendations for Interventional Trials (SPIRIT) checklist for this study protocol.

## 4 DISCUSSION

This study aims to design, execute and test a training intervention based on enhanced concepts of ST for managing FOC and choice of delivery method among nulliparous women. Based on the results of some studies that have investigated the relationship between FOC and SOC in pregnant women, women had less SOC with a strong FOC (Reducing all three components of a sense of cohesion) (Voogand et al., 2020), and the ability to cope with stress could increase a SOC in pregnant women (Mohamamdirizi et al., 2018).

Despite the integration of evidence about positive relationships between GRRs, SOC and parental outcomes during the perinatal period, evidence shows that ST is rarely used in pregnancy care research with healthy participants (Perez‐Botella et al., 2015). Therefore, it indicates the need for more in‐depth research into the underlying mechanisms of salutogenesis and its components to promote maternal health (Shorey & Ng, 2020).

Using ST in a few interventional studies on various health areas has produced promising results (Bonmatí‐Tomas et al., 2019; Heggdal & Lovaas, 2018; Odajima et al., 2017; Tan et al., 2016). Therefore, developing an effective intervention based on this theory focusing on promoting healthy behaviours can probably help women manage their FOC and reduce the costs of any potential consequences. This is precisely the opposite of designing interventions to address the consequences or causes. This intervention is the opposite of the pathogenesis approach, as the current study focuses on health promotion (Perez‐Botella et al., 2015; Shorey & Ng, 2020). The study findings prove the effectiveness. In that case, policymakers can employ it in developing public training strategies to support pregnant women and also by health staff in providing routine prenatal care to women.

Considering the health of most pregnant mothers and their infants during pregnancy (Hildingsson, 2017), the opportunity of reducing the FOC in pregnant women according to their different socio‐cultural characteristics (Nilsson et al., 2018) and determining the health status of individuals based on their mental experience in response to stressors (Ferguson & Davis, 2019), and potentials of ST in reducing FOC, these study goals to explain nulliparous women's experiences of FOC and review the determinants of FOC in nulliparous women to identify the main factors causing FOC and describe the concepts of ST in this regard. This study aims to develop a coping strategy based on the enhanced concepts of ST for managing FOC and encouraging vaginal delivery among nulliparous women.

### 4.1 Limitation

There is a possibility of low participation of nulliparous women due to the fear of contracting the COVID‐19 virus in the qualitative phase.

The review study will be limited to nulliparous women in Iran which is required to proceed with the next phase, a future clinical trial in this field.

Although we will try to include all eligible studies based on the aim of our review, some studies may be some studies will be lost unintentionally.

Nulliparous women, regardless of the FOC, are more willing to have a caesarean because their child is a golden baby, and they are concerned about their baby's health.

## 5 CONCLUSIONS AND IMPLICATIONS

Our study findings can be used to help nulliparous women better cope with childbirth‐related stresses and optimize the care provided to pregnant mothers to move towards greater health in the health/disease spectrum. Evaluating the programme's practical strategies can make pregnant women more inclined to have a vaginal delivery and prevent unnecessary caesarean section and its possible negative consequences. This programme can be the basis of efforts to promote the health of mothers in the future.

### TRIAL REGISTRATION

Iranian Registry of Clinical Trials: IRCT20210506051202N1. Registered August 21, 2021.

## AUTHOR CONTRIBUTIONS

The study protocol was designed by Safieh Kananikandeh, Farkhondeh Amin Shokravi and Mojgan Mirghafourvand, Farkhondeh Amin Shokravi, will provide supervision throughout the study as a principal investigator. Safieh Kananikandeh drafted the manuscript. Farkhondeh Amin Shokravi, Mojgan Mirghafourvand and Shayesteh Jahanfar. contributed to the critical revision of the manuscript. Safieh Kananikandeh will be responsible for participant recruitment, data collection and data management. Mojgan Mirghafourvand will contribute to randomization. Safieh Kananikandeh, Farkhondeh Amin Shokravi, Mojgan Mirghafourvand. and Shayesteh Jahanfar will contribute to the data analysis of the project. All authors have read and approved the final manuscript.

## FUNDING INFORMATION

This study is funded by Tarbiat Modares University in Iran (IR.Modares.RCE.1400.097). Research funders did not participate in research design, data collection, data analysis and publishing writing.

## CONFLICT OF INTEREST

The authors report no conflicts of interest.

## Appendix A

### A.1 DATA SET CATEGORY

**Table:**

Data set category	Information
Primary registry and trial identifying number	irct.ir IRCT20210506051202N1
Date of registration in primary registry	21 Aug, 2021
Secondary identifying numbers	–
Source(s) of monetary or material support	Tarbiat Modares University
Primary sponsor	Tarbiat Modares University
Secondary sponsor(s)	–
Contact for public queries	S.k., Ph.D Student of Health Education and Health Promotion [+984,143,344,231] [kanani.safieh@yahoo.com]
Contact for scientific queries	S.k., Ph.D Student of Health Education and Health Promotion [+984,143,344,231] [kanani.safieh@yahoo.com]
Public title	Saltuogenesis theory on fear of childbirth
Scientific title	Design, implementation, and evaluation of educational program based on saltuogenesis theory on fear of childbirth and choice of delivery method among nulliparous women: a sequential‐exploratory mixed methods design
Countries of recruitment	Iran
Health condition(s) or problem(s) studied	Fear of childbirth
Intervention	Educational content is prepared based on the main factors obtained from qualitative and review stages on the fear of childbirth in nulliparous women. The educational intervention is held in 4 training sessions of at least 30 min in 4 groups of 6 people and 2 groups of 7 people, one day a week for each group at the Health Comprehensive Service Centers of Bostanabad city of East Azerbaijan by the researcher in face to face. All of the training sessions will be completed by the 16th‐week gestation.
Key inclusion and exclusion criteria	Inclusion criteria: 18–35 years, High school and university, no history of self‐reported depression or long‐term illness, singleton pregnancy, ultrasound confirmation of foetal health, no history of miscarriage or stillbirth, lack of contraindications of vaginal delivery to termination of pregnancy, lack of midwifery base and related fields of study, having consent to participate in the study. Exclusion criteria: preterm delivery due to an emergency, indications for caesarean section, history of caesarean section, history of infertility, lack of continuous presence of pregnant women in training sessions, failure to complete the questionnaire, lack of access to the participant for any unpredictable reason.
Study type	Interventional Allocation: randomized Intervention model: parallel assignment Masking: Single blinded (assigned study groups.) Primary purpose: Education/Guidance
Date of first enrolment	Expectant
Target sample size	76
Recruitment status	Expected recruitment start date 23 Sep, 2022
Primary outcome(s)	Reduce fear of childbirth, Choice of natural childbirth
Key secondary outcomes	Sense of coherence

### A.2 INFORMED CONSENT FORM

**Informed Consent Form ‐ Ethics Committee in Biomedical Research, Tarbiat Modares University.**

Consent to participate in the project “Designing, Executing, and Evaluation of Educational Program Based on Salutogenesis Theory on Fear of Childbirth and Choice of Delivery Method Among Nulliparous Women: A Sequential‐Exploratory Mixed Methods Research Protocol.”

**Dear Mr./ Mrs. ..............**
You are hereby invited to participate in the above research. Information about this research is provided in this service sheet and you are free to participate or not to participate in this research. You do not have to make an immediate decision and to decide on this you can ask your questions to the research team and consult with anyone you want. Before confirming this consent form, make sure that you have understood all the information in this form and all your questions have been answered.
**Researcher.**

1. I know that the objectives of this study are:

‐ The study of fear of vaginal childbirth among primiparous women.

‐ The study on the choice of vaginal childbirth method among primiparous women.

‐ The study of the effect of a type of educational programme on fear of childbirth in primiparous women.

2. I know that participation is completely voluntary in this research.

3. I know that even after agreeing to participate in the research, I can leave the research whenever I want, after informing the researcher.

4. I have been assured that if I refuse to participate in this study or leave it at any stage after the initial agreement, I will not be deprived of routine diagnostic and therapeutic services.

5. How I collaborate in this research is as follows:

- Participate in a training programme on fear of childbirth and how to manage stress in pregnancy and childbirth among primiparous women.
- You will be asked about age, occupation, education, pregnancy, childbirth, information about childbirth, giving importance to the phenomenon of childbirth and stress management during pregnancy and childbirth.
- The information of clinical results in the personal file will be used.
- 76 nulliparous pregnant women will participate in this study.
- Cooperation in this study will continue until after delivery and registration of the type of delivery.
- During this period, 6 visits should probably be made. 5 times in the first trimester of pregnancy (probably once a week or once every 2 weeks) and 1 time 6 months after the fifth time.
- Each turn takes about 30 min.
- In the intervals of the second to the fifth referral, the items that are requested to focus on issues related to pregnancy and childbirth, such as family support during pregnancy, their role in the family, etc. and to take notes or even draw.
- The initial questionnaire is completed once again in the following.
- Upon request, we will be told which group we are in.
- Travel costs are the responsibility of the participant.

6. The potential benefits of my participation in this study are as follows

Participating in this study may be useful in identifying and controlling the source of stress and fear during pregnancy. In addition, participating in research can help improve the diagnostic and management methods of future pregnant women.

7. The possible harms and complications of participating in this study are as follows

- There is no opportunity of injury or complications.

8. If I do not want to participate in the study, I will be offered the usual treatment method, the benefits and side effects of which are as follows

- Routine pregnancy care in comprehensive healthcare centres does not provide services on stress and fear of childbirth and how to control and manage it.

9 I know that those involved in this research have kept all information about me confidential and are only allowed to publish the general and group results of this research without mentioning my name and details.
10 I know that the Research Ethics Committee can access my information to monitor compliance with my rights.
11 I know that I will not bear any of the costs of conducting the research as follows.
  ‐ Provide training programme.
  ‐ Training material.
  ‐ Workshop.
12 Ms. Safieh Kananikandeh was introduced to me to answer and I was told to share any problem or question regarding participating in the study with her and ask for guidance.

I was given her address and phone number as follows:

‐ Address: Valiasr Town, Bostan Abad, East Azarbaijan,

‐ Tel: 04143344231.

13 I know that if during and after the research any problem, both physical and mental, occurred to me due to participating in this research, the treatment of complications, and its costs and related compensation will be the responsibility of the researcher.
14 I know that if I have any problems or objections to those involved or the research process, I can contact the Research Ethics Committee at Tarbiat Modares University at Secretariat of the Ethics Committee, Faculty of Medical Sciences, Tarbiat Modares University, Jalal Al‐Ahmad Highway, and raise my problem orally or in writing with them.
15 This form of information and informed consent will be prepared in two copies and after signing, one copy will be given to me and the other copy will be given to the executor.

I have read and understood the above and based on it, I express my informed consent to participate in this research. Name and signature of the participant (if necessarily authorized surrogates). Farkhondeh Amin Shokravi consider myself obliged to fulfil the obligations related to the executor in the above provisions and I undertake to work to ensure the rights and safety of the participant in this research.
 Signature of the researcher.
